# A Sustainable Gel Polymer Electrolyte for Solid-State Electrochemical Devices

**DOI:** 10.3390/polym15143087

**Published:** 2023-07-19

**Authors:** Serena Tombolesi, Niccolò Zanieri, Luca Bargnesi, Martina Mernini, Giampaolo Lacarbonara, Catia Arbizzani

**Affiliations:** Department of Chemistry Giacomo Ciamician, University of Bologna, 40126 Bologna, Italy; serena.tombolesi2@unibo.it (S.T.);

**Keywords:** gel polymer electrolyte, capacitive electrode, sensors, actuators, poly vinyl alcohol, GPE

## Abstract

Nowadays, solid polymer electrolytes have attracted increasing attention for their wide electrochemical stability window, low cost, excellent processability, flexibility and low interfacial impedance. Specifically, gel polymer electrolytes (GPEs) are attractive substitutes for liquid ones due to their high ionic conductivity (10^−3^–10^−2^ S cm^−1^) at room temperature and solid-like dimensional stability with excellent flexibility. These characteristics make GPEs promising materials for electrochemical device applications, i.e., high-energy-density rechargeable batteries, supercapacitors, electrochromic displays, sensors, and actuators. The aim of this study is to demonstrate the viability of a sustainable GPE, prepared without using organic solvents or ionic liquids and with a simplified preparation route, that can substitute aqueous electrolytes in electrochemical devices operating at low voltages (up to 2 V). A polyvinyl alcohol (PVA)-based GPE has been cast from an aqueous solution and characterized with physicochemical and electrochemical methods. Its electrochemical stability has been assessed with capacitive electrodes in a supercapacitor configuration, and its good ionic conductivity and stability in the atmosphere in terms of water loss have been demonstrated. The feasibility of GPE in an electrochemical sensor configuration with a mediator embedded in an insulating polymer matrix (ferrocene/polyvinylidene difluoride system) has also been reported.

## 1. Introduction

Solid electrolytes have attracted much attention thanks to their wide application in batteries, supercapacitors, sensors, solar cells, and fuel cells [1,2,3,4,5,6]. The most important electrolyte requirements are: (i) high ionic conductivity, (ii) inertness towards the various species that may be present at the stage of assembly and/or resultant from the electrochemical or side reactions, (iii) reasonably low cost, (iv) stability in a relatively wide temperature range, and (v) chemical and electrochemical stability. The two main families of solid electrolytes are represented by inorganic-based and polymer-based electrolytes, with the presence of hybrid inorganic–polymer systems. Solid polymer electrolytes (SPEs), where the polymer can be synthetic or natural, offer more advantages over liquid electrolytes by being environmentally safe, flexible, and easy to handle. Organic liquid electrolytes have played an essential role in electrochemical energy storage for several decades due to their high ionic conductivities (10^−3^–10^−2^ S cm^−1^), wider electrochemical window compared to their aqueous analogues, and good interfacial contacts with electrodes [7]. In SPEs, there is no trace of solvent, and the ionic conductivity is due to the ion transport promoted by chain flexibility. Consequently, the specific conductivity at room temperature is usually in the range 10^−8^–10^−6^ S cm^−1^ and can increase significantly when the amorphization temperature of the polymer is reached. While it was advantageous to work with solid polymer electrolytes because they provide a promising opportunity to tackle the safety issue, the SPEs mostly display poor cycle performance due to their low ionic conductivity. To this end, combining the advantages of both liquid and solid electrolytes, gel polymer electrolytes (GPEs) have been demonstrated as excellent candidates for electrochemical devices such as batteries, supercapacitors, electrochromic displays, sensors, and actuators [8,9,10,11,12,13,14] thanks to their high ionic conductivity (close to liquid electrolytes) and solid-like dimensional stability with excellent flexibility.

Typically, in GPEs, a polymeric framework is utilized as the host material, ensuring strong mechanical integrity. The criteria to obtain a good polymer skeleton are: a (i) high molecular weight (10^4^–10^6^ g mol^−1^) for mechanical stability, (ii) low glass transition temperature (T_g_ < −30 °C) for fast segmental motion of the polymer chains, (iii) the presence of atoms or substituent groups promoting the dissolution of salts for ionic conductivity, (iv) a high degradation temperature (>200 °C), and (v) a wide electrochemical window for chemical and electrochemical stability (up to 5 V, depending on the solvent) [8]. Examples of host polymers include poly(ethylene oxide) (PEO), poly(vinylidene difluoride (PVdF) [15], poly(vinyl pyrrolidone) (PVP) [16], poly(acrylonitrile) (PAN) [17], poly(methyl methacrylate) (PMMA) [18] and poly(vinyl alcohol) (PVA) [19]. The salts are the source of charge carriers in GPEs and must have low dissociation energy to avoid ion pairs or aggregation. In addition, large anions produce a plasticizer effect. Thermal, chemical, and electrochemical stability are also required [20]. The selection of the solvent for the dissolution of polymer and salt is also crucial because the solvent remains entrapped in the final gel. In addition, a suitable solvent improves the ionic conductivity and determines the stability as well as the mechanical and chemical properties of GPE [9]. Among the organic electrolytes, cyclic carbonates or glymes are often used for devices requiring a wide electrochemical stability window. Ionic liquids may also provide excellent stability and good conductivity, avoiding the use of an organic solvent, even if they are quite expensive [21]. In contrast, for systems operating in a narrow voltage window, an aqueous environment could be indicated for the development of low-cost and safe devices [8]. 

This work aims to prepare and characterize a sustainable water-based GPE that can be used in several electrochemical devices. Polyvinyl alcohol was chosen as the polymeric matrix because of its excellent chemical stability, non-toxicity, biodegradability, water solubility and capability of incorporating large amounts of coordination water thanks to its -OH group, enhancing the electrolyte ionic conductivity [10]. Sodium chloride was selected as salt because it is non-toxic, stable, soluble in water and abundant. Also, glycerol was chosen as a plasticizer and supramolecular crosslinker for the GPE due to its non-toxicity, biodegradability, low volatility, and total miscibility in water [22]. The system PVA-glycerol-NaCl is not new; it has been prepared in different ways [23,24,25] and has been proposed for several applications, from tissue engineering applications to strain sensors and flexible supercapacitors [10,25,26]. In the present paper, a simplified preparation of the GPE has been proposed, avoiding the use of reflux systems or freezing/thawing processes, and directly adding the PVA to the aqueous solution of the NaCl for a better distribution of the salt between the polymer chains, with the addition of the plasticizer at the end. We performed tests to correlate the water loss of the GPE over time with the ionic conductivity in a closed and open environment. Indeed, the GPE can be used in sealed cells as well as in open devices. For this reason, the electrochemical properties of the GPE in two different device configurations have been tested. In a supercapacitor configuration, with capacitive electrodes, we tested the stability of the prepared GPE over a long operational life. In sensor configuration, with a redox mediator dispersed into a polymer insulating matrix, we tested the GPE reliability as an ionic conductor for devices open to the atmosphere. The latter characteristic could also be an added value for soft actuators. 

## 2. Materials and Methods

**Figure A1:**
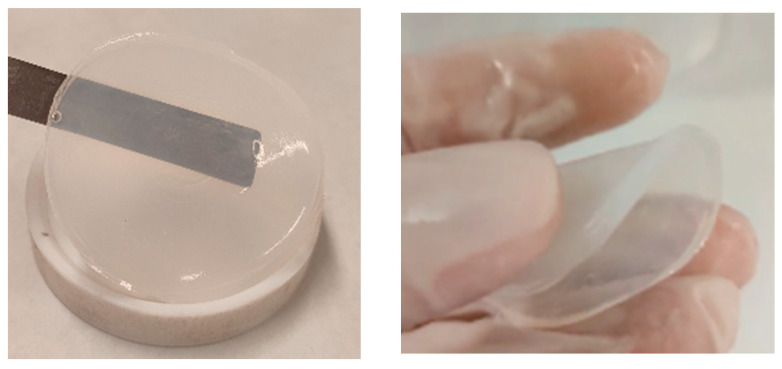
The as-prepared GPE (1.16 mm thick) with PVA:NaCl:glycerol:water = 1:0.5:1:5 weight ratios.

Polyvinyl alcohol (PVA, Sigma-Aldrich, Merk Life Science S.r.l., Milan, Italy, hydrolyzed, ≥99%, ρ = 1.269 g mL^−1^, T_m_ = 200 °C), sodium chloride (NaCl, Fluka, Merk Life Science S.r.l., Milan, Italy, ≥99.5%, MW = 58.44 g mol^−1^) and glycerol (Sigma-Aldrich, Merk Life Science S.r.l., Milan, Italy, ≥99.5%, MW = 92.09 g mol^−1^, T_b_ = 182 °C, ρ = 1.262 g mL^−1^) were used for the GPE preparation. The GPE was prepared with PVA:NaCl:glycerol:water weight ratios of 1:0.5:1:5, by dissolving NaCl and PVA in distilled water at 90 °C for 3.5 h under stirring. After having eliminated the bubbles by an ultrasonic bath treatment, glycerol was added and left under stirring at 90 °C for 30 min. The solution was poured into a Teflon mold and stored at room temperature for 16 h. The hydrogel thus obtained is homogeneous, transparent and very flexible. We obtained samples with a thickness ranging from 0.5 to 1.6 mm, depending on the amount of solution poured in the Teflon mold for casting the solvent (Appendix A, Figure A1). Thinner layers of GPE were yielded by direct casting on the electrode.

**Figure A2:**
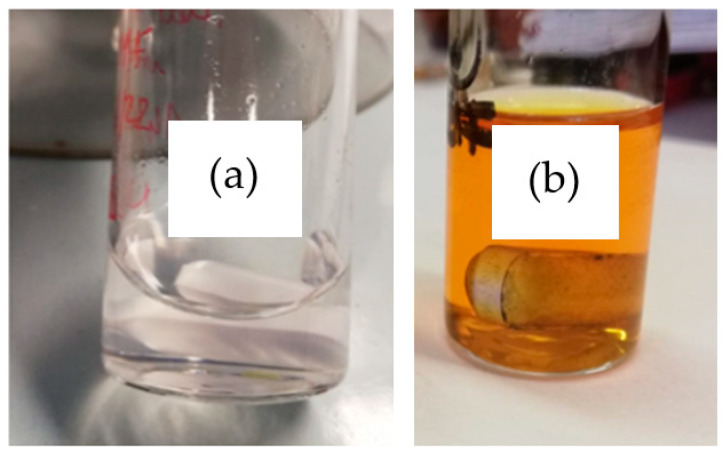
(**a**) PVdF in DMF solution; (**b**) PVdF + 20% wt. Ferrocene in DMF solution.

A model solid-state sensor with the redox mediator immobilized into an insulating polymer film was prepared from polyvinylene-difluoride (PVdF, Kynar (R) PVdF Arkema GmbH, Düsseldorf, Germany, HSV 900 PWD, ρ = 1780 kg/m^3^), dimethyl formamide (DMF, Alfa Aesar, Thermo Fisher GmbH, Kandel, Germany ≥99.8%, MW = 73.09 g mol^−1^, T_b_ = 153 °C, T_m_ = −61 °C and ρ = 0.944 g mL^−1^) and ferrocene (Fc, Aldrich Chemistry, Merk Life Science S.r.l., Milan, Italy, 98%, MW = 186.03 g mol^−1^). PVdF was dissolved in DMF and left under magnetic stirring overnight. Ferrocene (20% wt.) was added, and the solution was left under stirring for 3 h before being poured into a Teflon mold and kept at 80 °C to allow fast solvent evaporation. The homogeneous solution obtained was orangish (Appendix A, Figure A2). A thin film of PVdF-Fc was obtained by casting the solution on a conductive glass (ITO).

Activated carbon (AC, Picactif BP10 PICA USA, Inc. Columbus, Ohio, United States), 5 wt.% of conductive additive (Super C45, Imerys Graphite & Carbon Switzerland SA, Bodio, Switzerland) and 5 wt.% of polytetrafluoroethylene (PTFE) from aqueous suspension (Dupont De Nemour Netherlands B.V., Dordrecht, Netherlands, 60 wt.%) were used to prepare AC electrodes by grinding the solids in a mortar and adding stepwise 100 µL of ethanol (Sigma Aldrich, Merk Life Science S.r.l., Milan, Italy, absolute ≥ 99.8%). Self-standing electrodes were laminated until a uniform thickness was obtained. The electrodes (9 mm diameter, 3–10 mg cm^−2^) were punched and dried under a vacuum at room temperature (RT) overnight.

Several methodologies were used to assess the water retention of the GPE by varying the time, temperature and humidity. Thermogravimetric analysis (TGA) was carried out with a Q50 TA Instrument (Waters S.p.A., Milan, Italy), and electrochemical impedance spectroscopy (EIS) with a VSP potentiostat/galvanostat (BioLogic SAS, Seyssinet-Pariset, France). FTIR-ATR (Bruker ALPHA FTIR spectrometer, Milan Italy) was used to evaluate the GPE and its single components between 400 and 4000 cm^−1^, 64 scans, at RT. Differential scanning calorimetry (DSC) was carried out using a Q2000 DSC apparatus (TA Instruments, Waters S.p.A., Milan, Italy) equipped with a refrigerated cooling system (RCS90). About 8–10 mg of sample was placed in hermetic aluminum pans and subjected to a heating scan at 20 °C min^−1^ from −40 °C to +90 °C, quenched to −40 °C, and then heated up to 90 °C at 20 °C min^−1^, under a nitrogen atmosphere. From the acquired data, the glass transition temperature (T_g_) could not be determined. The humidity of the atmosphere was measured with a Trotec BC21 hygrometer (Trotec GmbH & Co. KG, Heinsberg, Germany). Electrochemical tests were carried out using different setups with a VSP potentiostat/galvanostat (BioLogic SAS, Seyssinet-Pariset, France): T-shaped Teflon cells (Bola, Bohlender GmbH, Grünsfeld, Germany) with stainless steel plugs as current collectors were used for measuring the ionic conductivity by impedance spectroscopy. Cells with titanium discs or grids as collectors were used for cyclic voltammetry and electrochemical stability tests in supercapacitor configuration. The specific currents of the galvanostatic charge/discharge cycles refer to the mass of both electrodes (m_d_). The cell for tests in sensor configuration is described in Section 3.3.

## 3. Results

### 3.1. Physicochemical Characterization

A PVA:NaCl:glycerol:water weight ratio of 1:0.5:1:5 was selected. Formulations with higher salt concentrations (1:1:1:5 and 1:0.8:1:5) did not seem to be viable with the proposed simplified procedure. The obtained GPEs were rigid and, apparently, with crystallized parts. 

**Figure 1:**
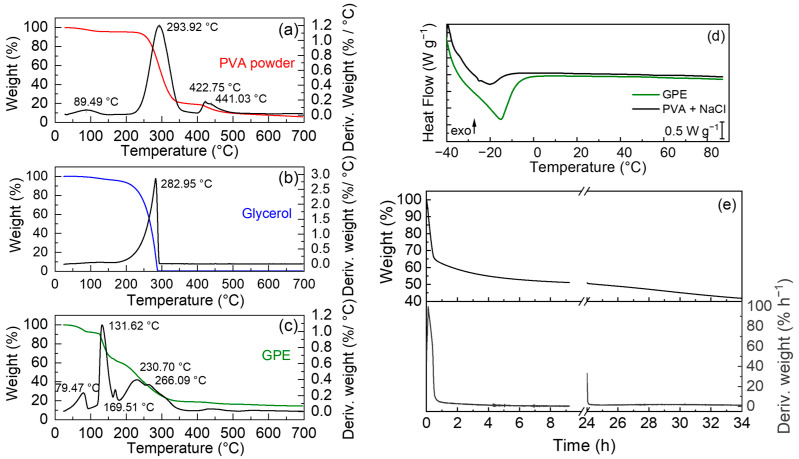
TGA performed in Ar at 10 °C min^−1^ from RT to 700 °C of (**a**) PVA powder, (**b**) glycerol, and (**c**) GPE; (**d**) DSC first heating scan of GPE (green) and PVA + NaCl (black); (**e**) TGA of freshly prepared and 24 h-aged GPEs performed at 30 °C in Ar:O_2_ (80:20 mL min^−1^).

The thermogravimetric analysis of GPE was performed in Ar from RT to 700 °C with a heating rate at 10 °C min^−1^, as shown in Figure 1a–c. The GPE displays a first step of degradation at 79 °C, due to the free water loss [27]. A second degradation step is visible at 131 °C, due to coordination water loss; then, between 230 and 270 °C, there are different degradations ascribed to the decompositions of PVA and glycerol that are also affected by the interactions between them. This first step of degradation of PVA is then followed by degradation in the second last step at 420 °C.

DSC analyses were carried out on GPE and on a PVA + NaCl gel (without glycerol) prepared with the same procedure described in Section 2. The calorimetric curves show an endothermic peak around −20 °C that can be attributed to the presence of water in the hosting structure [25]. The supramolecular crosslinking with glycerol increases the enthalpy of the process (from 12 to 59 J g^−1^), suggesting more interactions between water molecules and the polymer structure of the GPE with respect to only PVA (Figure 1d).

To evaluate the solvent loss from GPE over time, an isothermal thermogravimetric analysis was performed over 9 h at 30 °C in a mixture of argon and oxygen, 80 mL min^−1^ and 20 mL min^−1^, respectively, (Figure 1e) in order to investigate changes in the sample weight in conditions mimicking the atmosphere. Two GPE samples at different times from the preparation were analyzed: one as prepared and one aged for 24 h in air. The curve of the aged GPE was then shifted and combined to the curve of the GPE as prepared. The rate of weight loss over time is calculated during the first 40 min, and the GPE water loss is significant (ca. 30%), which could be attributed to the free water present in the system. Furthermore, the rate of weight loss stabilizes between 0.5 and 1% h^−1^, with an additional 15% weight loss in the remaining time, indicating the slower evaporation rate of the coordinated water. To assess whether this water loss impacts the conductivity, EIS spectra were carried out over time in the Teflon T-shaped cell. 

**Figure A3:**
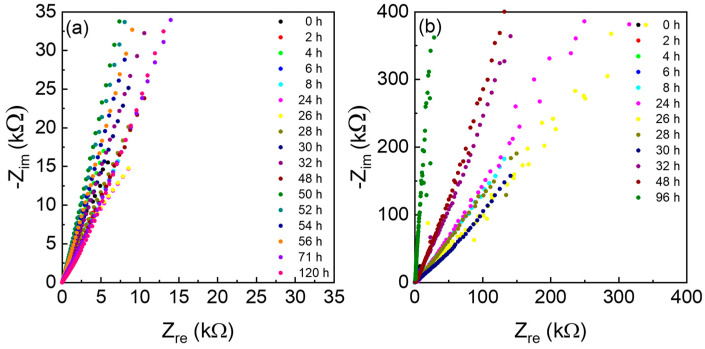
EIS spectra at different times (**a**) in a sealed cell SS/GPE (1.42 mm)/SS and (**b**) in open atmosphere GPE (1.57 mm) from 200 kHz to 1 Hz.

Impedance spectra of cells with the GPE placed between blocking stainless steel electrodes were collected over 96 or 120 h with 5 mV (AC) and in the 200 kHz–1 Hz frequency range at RT (Appendix A, Figure A3). Given that water loss likely affects the GPE thickness, the cell was disassembled after each EIS test, and the GPE thickness and diameter were measured, the former with a digital micrometer and the latter with ImageJ software. The GPE was maintained in the sealed cell for the resting time between two measurements. A similar experiment was carried out by maintaining the GPE in air, covered by a plastic box to protect it from dust, and by placing it in the cell only for the EIS test.

**Figure 2:**
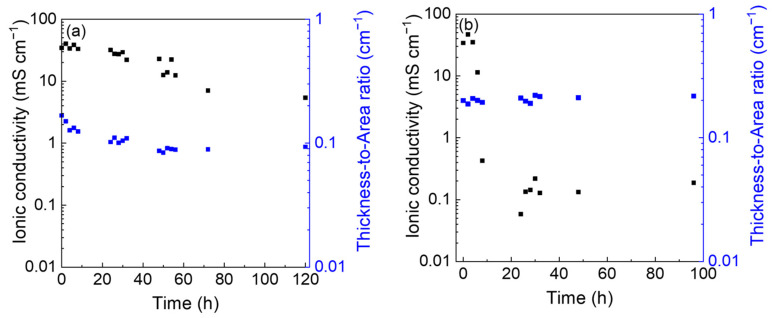
Ionic conductivity and thickness-to-area ratio of GPE over time at RT (**a**) in a sealed cell AC/GPE (1.42 mm)/AC and (**b**) in open atmosphere GPE (1.57 mm).

Hence, the ionic conductivity was calculated by the formula σ = l/RA with l as the thickness, R as the resistance, and A as the area of the GPE. Figure 2a shows the EIS spectra performed at different times at RT, while Figure 2b displays the ionic conductivity and the thickness of the GPE over time. The resistance, thickness, and ionic conductivity values of GPE are reported in Appendix A, Table A1. 

The conductivity trend obtained in a sealed cell shows that the GPE is stable in these conditions with a conductivity that slightly decrease from 35 mS cm^−1^ to 5 mS cm^−1^ in 120 h (Figure 2a). It must be considered that the sample manipulation needed to perform ex situ measurements of the thickness and the area could accelerate the water loss with a correspondent decrease in the ionic conductivity. On the other hand, in the open atmosphere, the GPE conductivity shows a steep drop over time due to faster water evaporation (Figure 2b). After 24 h, the ionic conductivity stabilizes around 10^−1^ mS cm^−1^.

The ionic conductivity was also measured as a function of the air humidity (Table 1). In devices operating in open atmosphere, the humidity could influence the water loss of the GPE and, thus, the eventual conductivity variation. Different samples were left in a closed glass container under atmosphere with controlled humidity (10%, 50%, 90%) for 4 h and their conductivity measured by EIS in SS/GPE/SS cell. All the samples showed an initial conductivity between 34 and 36 mS cm^−1^.

**Figure 3:**
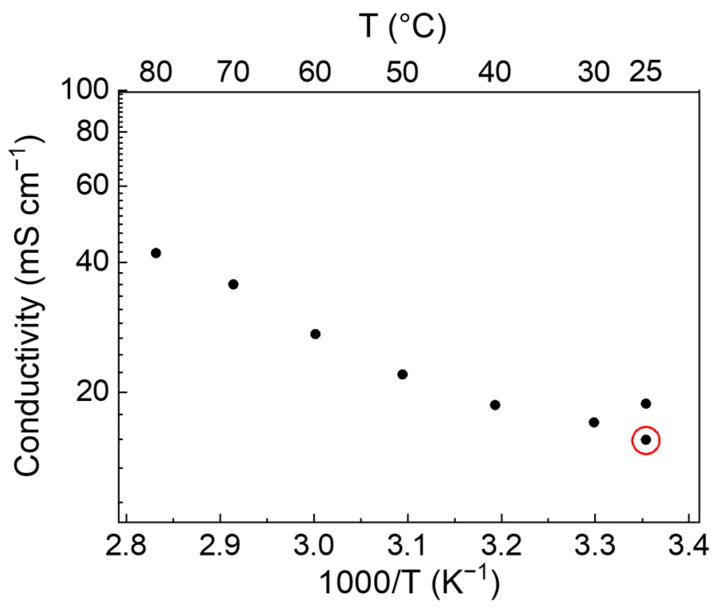
Conductivity data between 25 °C and 80 °C from EIS spectra of a symmetric AC/GPE (1.16 mm)/AC (electrode active mass 5.8 mg cm^−2^ each) cell. The red circle indicates the measure when the cell spontaneously cooled at 30 °C after having concluded the heating ramp up to 80 °C.

**Figure A4:**
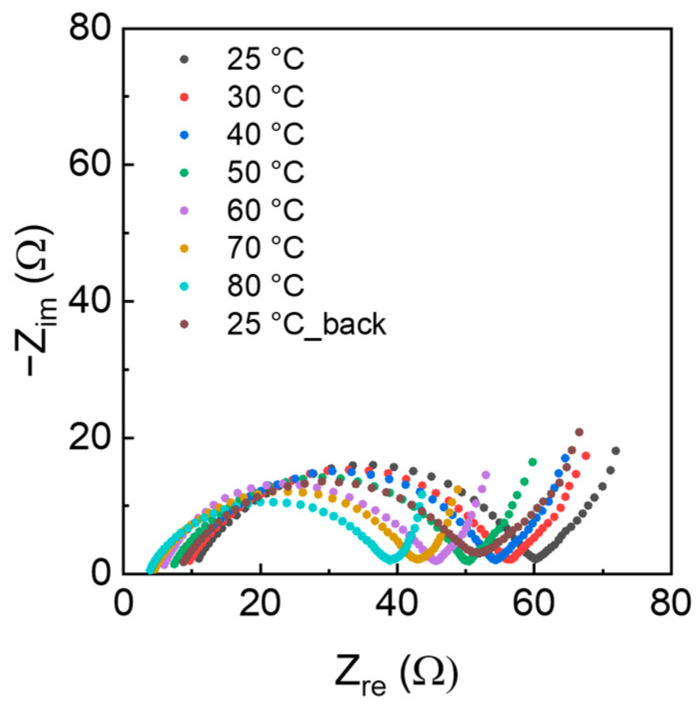
EIS spectra at different temperature from 100 kHz to 100 mHz of a symmetric AC/GPE (1.16 mm)/AC (electrode active mass 5.8 mg cm^−2^ each).

From EIS spectra of the cell with AC electrodes, reported in Appendix A, Figure A4, the conductivity values of the GPE have been evaluated at different temperatures, after 1 h resting at each temperature, and are reported in Figure 3. The electrolyte resistance has been evaluated by the intercept at high frequency of the semicircle. The conductivity increases, as expected, with temperature even if there is no significant variation between 25 °C and 80 °C, where the conductivity is less than three times higher than at 25 °C. Figure 3 also reports the value at 25 °C, recorded after 48 h when the ramp up to 80 °C was concluded, and the cell naturally cooled (red circled point). The higher conductivity can be associated with rearrangements of the chains and ions in the GPE. From the Arrhenius plot, a low activation energy of 0.23 kJ mol^−1^ (2.3 meV) can be evaluated, indicating good mobility of ions in the GPE.

**Figure 4:**
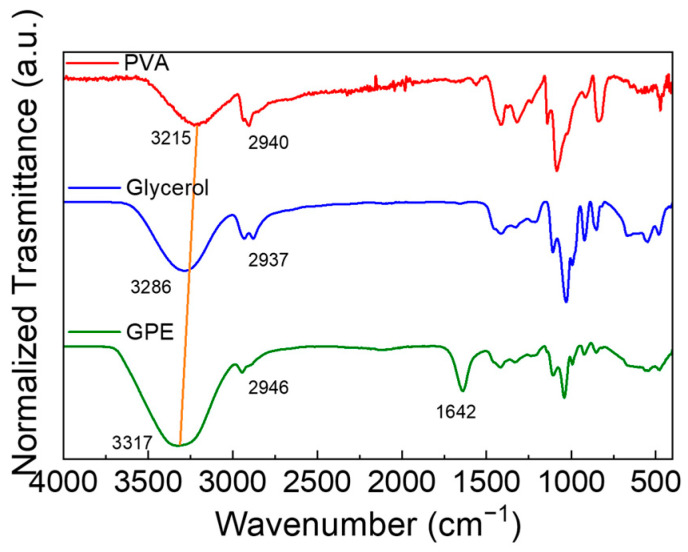
FTIR-ATR spectra of PVA (red), glycerol (blue), and GPE (green).

Gel polymer electrolyte has also been characterized by FTIR-ATR spectroscopy. Figure 4 shows the spectra of PVA, glycerol and GPE. In the pure PVA spectrum, the O-H stretching is present at 3200 cm^−1^. A less intense vibration at 2940 cm^−1^ is due to the asymmetric stretching of the C-H bond [28]. The pure glycerol O-H stretching is present at 3281 cm^−1^, which is more intense than that present in the PVA spectrum, given it is a triol. Another peak is around 2950 cm^−1^ due to the asymmetric stretching of the C-H bond. Finally, in the spectra of GPE, the vibrations due to the stretching of the O-H bond are centered at 3317 cm^−1^, and the C-H bond slightly shifted at a higher wave number with respect to those of glycerol. This is mainly ascribed to the coordination water in the GPE, which cannot be removed by a mild drying procedure (as shown in the TGA of Figure 1). The presence of water is also confirmed by the board band at 2105 cm^−1^ and the vibration at 1642 cm^−1^ [29]. 

### 3.2. Electrochemical Tests with AC Electrodes

**Figure 5:**
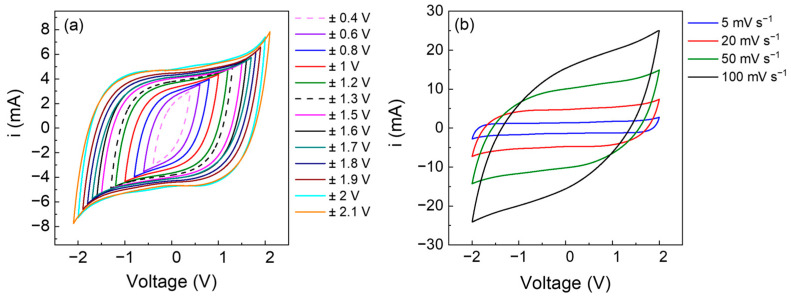
CVs at 20 mV s^−1^ of AC/GPE (1.16 mm)/AC cell (electrode active mass 6.8 mg cm^−2^) (**a**) in different voltage windows and (**b**) at different scan rates: 5 mV s^−1^ (blue), 20 mV s^−1^ (red), 50 mV s^−1^ (green), and 100 mV s^−1^ (black).

Electrochemical measurements were performed in a cell with titanium current collectors to avoid unwanted reactions, and applying a pressure of ca. 4.7 kg cm^−2^, i.e., 4.6·10^5^ Pa, to improve the contact between the electrode and the electrolyte. Two-electrode symmetric cells were assembled using two activated carbon electrodes and the GPEs as electrolytes. Cyclic voltammetries (CVs) were first performed at 20 mV s^−1^, varying the potential window up to ±2.1 V (Figure 5a). Then, subsequent CVs were carried out at different scan rates: 5, 20, 50, and 100 mV s^−1^ (Figure 5b). CVs display a typical capacitive behavior with well-defined box-shaped cycles. 

**Figure 6:**
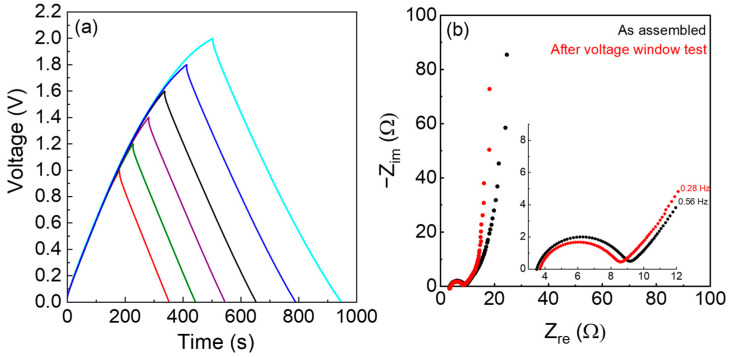
AC/GPE (0.703 mm)/AC cell (electrode active mass 9.7 mg cm^−2^). (**a**) Voltage profiles at 0.1 A g^−1^; (**b**) EIS spectra from 100 kHz to 10 mHz of the AC/GPE (0.703 mm)/AC cell as assembled (black) and after the galvanostatic cycles (red). The enlarged high-frequency region is in the inset.

The electrochemical performance of the GPE was also evaluated by galvanostatic charge and discharge cycles in different currents and voltage ranges. Figure 6a displays the profiles at 0.1 A g^−1^, and Figure 6b shows the EIS spectra carried out before and after the galvanostatic cycles. The voltage profiles of Figure 6a are typical of a supercapacitor, and it is worth noting that the voltage window is higher compared to water-based electrolytes with conventional concentrations of salts.

The EIS spectra of Figure 6b were fitted with the circuit R_e_(RQ)WQ_L_, because it was possible to identify the electrolyte resistance as the intercept at high frequency (R_e_) of the semicircle. The semicircle originates from the charge transfer resistance (R) in parallel to the related double layer capacitance (Q). At low frequency, a Warburg element (W) and the capacitance of the device Q_L_ is visible. The phase constant element Q was used for the fitting, instead of the capacitance accounting for the reality of the system.

The electrolyte resistance of the freshly assembled cells was 3.6 ± 0.1 Ω and 3.8 ± 0.4 Ω after the galvanostatic cycles. The corresponding electrolyte ionic conductivity is 24 mS cm^−1^. The equivalent series resistance, which can be evaluated both from the intercept of the semicircle at low frequency and from the ohmic drop of the galvanostatic curves, is in the order of 9.0 ± 0.5 Ω, i.e., 14.9 Ω cm^2^.

**Figure 7:**
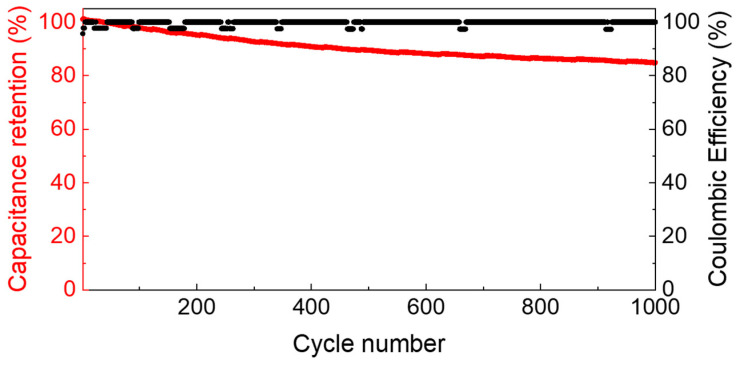
Electrode capacitance retention (red) and coulombic efficiency (black) in an AC/GPE (1.16 mm)/AC supercapacitor at 0.5 A g^−1^ at RT (electrode mass loading 6.8 mg cm^−2^).

Repeated charge and discharge tests at 0.5 A/g demonstrated that the GPE is stable over cycling, as reported in Figure 7, where the capacitance retention and the coulombic efficiency are plotted vs. the cycle number. The capacitance retention was evaluated as the ratio between the discharge capacitance at a certain cycle and the discharge capacitance of the first cycle. The coulombic efficiency is the percentage ratio between the discharge capacity and the charge capacity. The capacity (Q_e_, in C), the capacitance (C_e_, in F) and the coulombic efficiency percentage (η%) of the single electrode were evaluated by

Q_e_ = ∫I dt/m_e_ (1)

C_e_ = 4 (I dt/dV)/m_d_ (2)

η% = Q_e,disch_/Q_e,ch_ (3)

where I (A) is the discharge current, t (s) is the discharge time, m_e_ and m_d_ (g) are the mass of the active material of one electrode or both electrodes of the device, respectively, and V (V) is the discharge voltage [30].

### 3.3. Electrochemical Tests with PVdF + 20% wt. Fc Electrode

**Figure 8:**
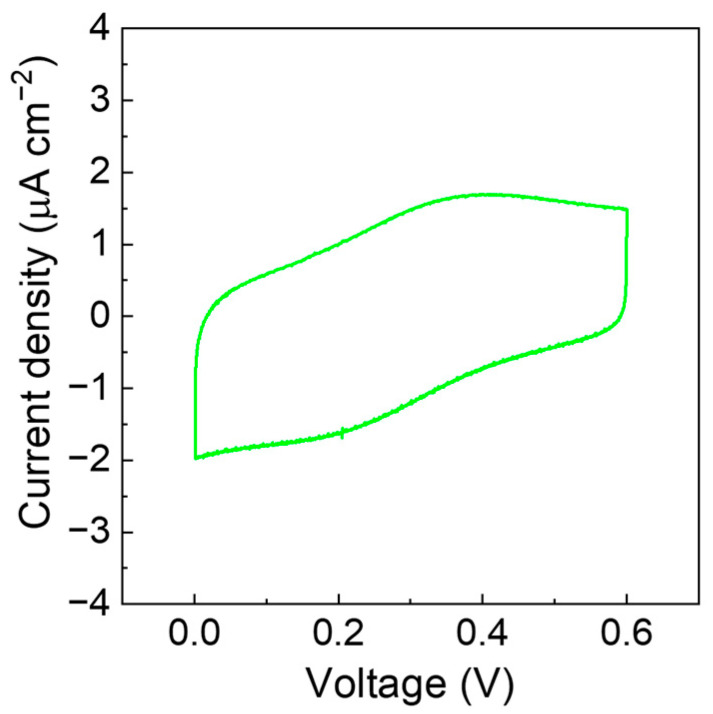
CV of ITO/GPE (0.013 mm)/PVdF-Fc (0.084 mm) performed at RT at 100 mV s^−1^ where the signal of ITO/GPE (0.013 mm)/ITO cell has been subtracted.

**Figure A5:**
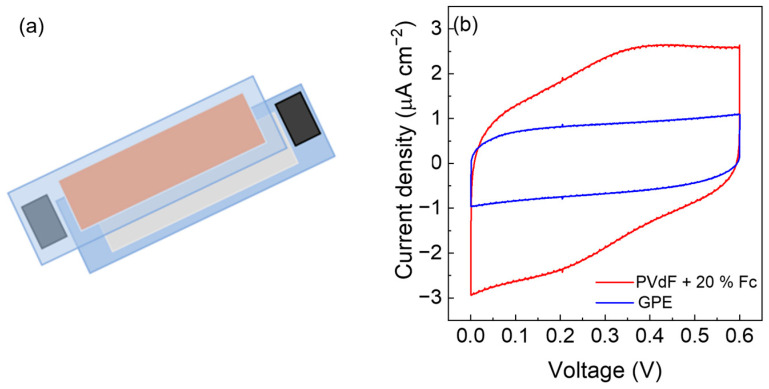
(**a**) Cell setup used to perform electrochemical test on GPE and PVdF + 20% wt. Fc films. The working and counter electrode contacts were taken using platinum foils. (**b**) CVs at RT and 100 mV s^−1^ of the ITO/GPE (0.013 mm)/PVdF-Fc (0.084 mm) (red) and of the ITO/GPE (0.013 mm)/ITO (blue).

Electrochemical tests on electrodes where the electroactive species is entrapped into an insulating polymer matrix were carried out to assess the applicability of the GPE in sensors, as well as in electrochemical soft actuators. A thin layer of PVdF + 20% wt. Fc solution in DMF was cast on an ITO glass (area = 0.72 cm^2^) and dried at 80 °C for 15 min. The film thickness was 0.084 mm. On another ITO glass, a thin layer of GPE solution was cast (area = 2.2 cm^2^), with a thickness of ca. 0.013 mm. The two ITO glasses were then assembled as in Appendix A, Figure A5a. CVs were performed on an assembled ITO/GPE/PVdF-Fc device and on ITO/GPE/ITO, both displayed in Figure A5b. To evidence the redox process of Fc, the capacitive response of an ITO/GPE/ITO system was subtracted to the CVs of the ITO/GPE/PVdF-Fc cell. Figure 8 shows the CVs of the device after the response of GPE has been subtracted to better evidence the redox process of Fc. 

## 4. Discussion

The PVA-based GPE, prepared from a simplified water casting route, displayed good mechanical, thermal and electrochemical properties. The TGA measurements reported in Figure 1a show the degradation steps of PVA, glycerol, GPE, and water losses. In the TGA plot related to the GPE, a first peak is observed at 79 °C, which is attributed to an initial water loss of 8% wt., due to free water still present in the polymer matrix. A second weight loss step of 32% wt. is observed at around 131 °C, attributed to the loss of water coordinated in the GPE with PVA and glycerol, forming H-bonds. 

Two peaks in the 230–267 °C range are attributed to the degradation of the glycerol and PVA, respectively. For PVA, the degradation occurs due to the elimination of hydroxyl groups as water, chain-scission, and the formation of double bonds in the structure. Glycerol degradation leads to decomposition with the formation of volatile components, which are carried away by the argon flow [31,32]. It must be noted that in the GPE, the decomposition of these components results shifted at lower temperatures because of the weaker H-bond hetero-interactions between PVA and glycerol with respect to the homo-interactions present in the pure compounds. At 432 °C, one last step of degradation occurs, which is attributed to the residue of the initial PVA. These residues contribute to thermal degradation and possess similar structures, such as conjugated structures and carbonyl groups. Isothermal thermogravimetric analysis evidenced two different water evaporation rates, suggesting that after the fast free-water evaporation, the coordinated water remain more constrained in the GPE contributing to increase the ionic conductivity.

The conductivity values of the as-prepared GPEs are around 33–36 mS cm^−1^ at room temperature, whose results are comparable with samples obtained using a reflux procedure. Peng et al. reported a conductivity of 46.8 mS cm^−1^ for a GPE composition of 1:0.6:1:7 ratios, and they obtained higher conductivity values for the 1:1.4:1:7 formulation (92.5 mS cm^−1^) [23]. However, it was difficult to reach such a high concentration of salt by simply mixing the solution, as in our case. Nevertheless, the ionic conductivity of the GPE is satisfactorily high even at this low salt concentration and shows an Arrhenius-type behavior with a low activation energy (2.3 meV) indicating a small energy barrier for ion transport. As expected, in open systems, the ionic conductivity is dependent on the air humidity due to the different water evaporation rates, ranging from 0.1 to 35 mS cm^−1^ after 4 h of air exposure at 10 and 90% humidity, respectively.

Furthermore, this problem is minimized in sealed devices or systems that could have brief contact with the atmosphere (up to a few hours). From an electrochemical point of view, GPE properties have been evaluated in a sealed system. The configuration was the same as a solid-state supercapacitor with activated carbon electrodes. Despite the high thickness of the GPE, the device works in a relatively wide voltage range of up to 2 V, with box-shaped CVs and linear charge and discharge voltage profiles. The electrode capacitance was ca. 90 F g^−1^ at the lowest current density, 1 mA cm^−2^, i.e., 0.1 A g^−1^, and decreased at ca. 70 F g^−1^ at 5 mA cm^−2^, i.e., 0.5 A g^−1^. The ohmic drop values are aligned with those reported for other solid-state systems with GPE [10]. The electrochemical stability, evaluated in terms of capacitance retention over 1000 cycles at 0.5 A g^−1^, is good (85%) and evidence that the electrolyte is electrochemically stable. Also, the coulombic efficiency, very near 100%, indicates the good electrochemical stability of the system. For all these reasons, the GPE is an interesting solid electrolyte for electrochemical devices like supercapacitors or Li ion batteries and Na ion batteries that can operate in an aqueous medium, providing suitable selection of the salt. 

Another field of application that can take advantage of this kind of electrolyte is that of sensors [33]. The experiment has been designed to determine if the electroactive molecule, embedded in an insulating matrix (here Fc in PVdF), can be electrochemically stimulated in a solid-state configuration with the GPE as a solid electrolyte. In this case, we used a thin layer (<100 μm) of electrolyte directly cast on the PVdF-Fc film. In this configuration, the redox behavior of ferrocene was observed in the selected electrochemical window, from 0 V to 0.6 V. This is a promising result for the GPE, which could pave the way for application in other systems, like electrochemical soft actuators that need solid-state configurations but, at the same time, can operate in aqueous environments. 

## 5. Conclusions

A sustainable GPE has been prepared with low-cost and abundant components, and by easy processing. It exhibits good mechanical, thermal and electrochemical properties, suitable for several electrochemical devices. The ionic conductivity was related to the water retention of the GPE in different conditions (resting time, temperature, and humidity) and at RT ranging from 35 mS cm^−1^ of the as prepared sample to 0.1 mS cm^−1^ of the sample stored in air for 4 h at 10% humidity. The first, close device allowed us to demonstrate the good performance of the GPE. After 1000 cycles at a high specific current (0.5 A g^−1^), the capacitance retention is 85% with a coulombic efficiency near 100%. The second device, a model sensor, indicates that a thin layer of GPE allows the circuit to be closed in which the working electrode is covered by PVdF embedding ferrocene, the electroactive species that mimics a redox mediator. We activated the redox mediator dispersed into a polymeric insulating matrix using the water-based gel polymer electrolyte. In this system, the electrochemical stimulus is transferred to ferrocene that can be reversibly switched from the oxidized to the reduced state and a current flow in the device. With this approach, we can also use the GPE for electrochemically stimulated soft actuators.

## Figures and Tables

**Table 1:** Ionic conductivity values as function of the humidity measured at RT after 4 h.

Humidity	Conductivity (mS cm^−1^)
10%	0.24
50%	3.65
90%	35.65

## Data Availability

The data presented in this study are available on request from the corresponding author.

## Appendix A

**Table A1:** Resistance (from Figure A3), measured thickness and diameter, and calculated ionic conductivity of GPE (reported in Figure 2) stored in a sealed cell and in open atmosphere at RT.

Time (h)	Resistance (Ω)		Thickness (µm)		Diameter (mm)		Conductivity (mS cm^−1^)	
	Sealed	Open	Sealed	Open	Sealed	Open	Sealed	Open
0	4.9	5.9	1419	1568	1.04	1.00	35	34
2	3.7	4.0	1252	1441	1.03	0.99	40	47
4	3.7	5.9	1184	1320	1.09	0.90	34	35
6	3.4	18	1127	1137	1.04	0.85	38	11
8	3.7	454	1055	1046	1.04	0.83	33	0.43
24	3.2	3588	901	1025	1.06	0.79	32	0.06
26	4.0	1467	868	1018	1.00	0.81	28	0.13
28	3.7	1318	870	1000	1.05	0.82	27	0.14
30	3.6	1009	822	972	1.00	0.75	29	0.22
32	4.9	1679	776	1004	0.95	0.77	22	0.13
48	3.8	1587	736	982	1.04	0.77	23	0.13
50	6.7	-	739	-	1.06	-	12	-
52	6.6	-	729	-	1.01	-	14	-
54	4.0	-	731	-	1.02	-	22	-
56	7.1	-	724	-	1.02	-	12	-
72	12.6	-	715	-	1.01	-	7	-
96	-	1156	-	960	-	0.75	-	0.19
120	17.3	-	704	-	0.98	-	5	-
